# Prognostic value of molecular cytology by one-step nucleic acid amplification (OSNA) assay of peritoneal washings in advanced gastric cancer patients

**DOI:** 10.1038/s41598-022-16761-8

**Published:** 2022-07-21

**Authors:** Katarzyna Gęca, Magdalena Skórzewska, Karol Rawicz-Pruszyński, Radosław Mlak, Katarzyna Sędłak, Zuzanna Pelc, Teresa Małecka-Massalska, Wojciech P. Polkowski

**Affiliations:** 1 Department of Surgical Oncology, Medical University of Lublin, Radziwiłłowska 13 St., 20-080 Lublin, Poland; 2 Department of Human Physiology, Medical University of Lublin, Radziwiłłowska 11 St., 20-080 Lublin, Poland

**Keywords:** Molecular biology, Cancer, Gastrointestinal cancer, Metastasis, Tumour biomarkers

## Abstract

Peritoneal dissemination is a common form of gastric cancer (GC) recurrence, despite surgery with curative intent. This study aimed to evaluate the prognostic value of intraperitoneal lavage One-Step Nucleic Acid Amplification (OSNA) assay in advanced GC patients. OSNA assay targeting CK-19 mRNA was applied to detect free cancer cells (FCC) in intraperitoneal lavage samples obtained during gastrectomy. A total of 82 GC patients were enrolled to investigate the correlation between OSNA assay and patient’s prognosis. Of the 82 patients, OSNA assay was positive in 25 (30.5%) patients. The median OS in OSNA positive patients was significantly lower than in OSNA negative patients (19 vs 45 months). Positive OSNA assay result was a significant unfavourable prognostic factor in both, univariable (HR 3.45, 95% CI 0.95–12.48; p = 0.0030) and multivariable analysis (HR 3.10, 95% CI 1.22–8.54; p = 0.0298). Positive OSNA assay in intraperitoneal lavage is a valuable indicator of poor survival in advanced GC patients after multimodal treatment. After further confirmation on larger sample size, OSNA assay of peritoneal washings could be considered an adjunct tool to conventional cytology, the current gold standard, to provide precise intraoperative staging and additional prognostic information.

## Introduction

Although the incidence of gastric cancer (GC) has steadily declined over the past 50 years, it remains one of the most common and deadly cancers^1^. Worldwide, more than 1 million new cases and an estimated 783 000 deaths caused by GC were recorded in 2018, making it the fifth most frequently diagnosed cancer and the third-leading cause of cancer death^2^. Despite surgery with curative intent, peritoneal dissemination is a common form of GC recurrence^3^. It is believed that the cause of spread may be due to the free cancer cells (FCC) present in the peritoneal cavity during surgery^4^. Although the presence of FCC is associated with advanced GC stage and poor prognosis, there is no standard treatment for patients with microscopic peritoneal dissemination^3^. The REGATTA trial demonstrated that limited gastrectomy followed by systemic chemotherapy (CTH) did not affect survival compared with CTH alone in patients with peritoneal metastases from GC^5^. Japanese GC treatment guidelines 2018 (5th edition) recommend CTH for peritoneal washings cytology-positive (CY1) patients who underwent gastrectomy^6^. Furthermore, there is no agreement on the routine testing for FCC in peritoneal fluid, the methods of detection, and the relationship of FCC to the clinical and pathological variables^3^.

Determination of the clinical stage of GC at baseline provides the necessary information to develop an appropriate treatment strategy^7^. Computed tomography (CT) has good accuracy in GC staging, but sensitivity and specificity for detection of peritoneal metastases remain poor^8^. The utility of staging laparoscopy (SL) for preoperative staging of GC has been proposed in many studies^9,10^. It has been suggested that SL has better sensitivity, specificity and accuracy than other imaging modalities, especially in detecting peritoneal metastases (87%, 100% and 91%, respectively)^9,11^. SL with peritoneal washings enables detection of FCC by cytology^12^. According to the 8th edition of the Tumour, Node and Metastasis classification (TNM), patients with CY1 are classified as M1, a stage IV of GC^13^. The European Society for Medical Oncology (ESMO) recommendation is to perform the peritoneal washings analysis in all patients with a potentially resectable GC (stages IB–III)^14^.

Out of many peritoneal washings examination methods, conventional cytology with Papanicolau or Giemsa staining is most common^14,15^. Immunohistochemical (IHC) methods based on the reaction of antibodies against antigens presented on tumour cells improve cytological sensitivity^10^. Molecular diagnostics using RT-PCR has also been used to detect FCC in peritoneal fluid due to its high sensitivity^16^. The sensitivity and specificity of all these methods for predicting peritoneal recurrence vary widely^14^. The conventional cytology remains the standard for peritoneal washings examination due to its simplicity^17^. The cytology’s sensitivity, specificity and overall accuracy in predicting peritoneal recurrence is 11–80%, 86–100% and 73–92%, respectively^15^. Due to the relatively low sensitivity of conventional cytology, many patients with negative cytology (CY0) may experience unexpected and rapid peritoneal recurrence after surgery performed with curative intent^12^.

It has been reported that molecular detection of tumour markers such as carcinoembryonic antigen (CEA), cytokeratin 19 (CK-19), cytokeratin 20 (CK-20) is better correlated with peritoneal recurrence and associated with adverse outcomes in patients with GC^10,17–19^. It has been proven that CK-19 may serve as a suitable marker of metastases in GC patients. CK-19 is commonly expressed by cancer cells of epithelial origin but not by lymphoid or hematopoietic cells^20^. It has also been reported that CK-19 may exceed CK-20 in detecting circulating cancer cells in peripheral blood from GC patients by means of RT-PCR^21^. Results of the research demonstrate that CK19 mRNA detected by reverse transcription-loop-mediated isothermal amplification (RT-LAMP) may be suitable as an intraoperative diagnostic modality for detecting GC patients with high risk of recurrence even after clinically curative surgery who require appropriate adjuvant treatment^22^. A meta-analysis demonstrated that molecular analysis of peritoneal fluid could be a prognostic factor for patients with negative cytological findings and/or receiving curative treatment^23^.

As we previously described, One-Step Nucleic acid Amplification (OSNA) can be applied for fast detection of CK-19 mRNA in peritoneal washings, reflecting the amount of FCC in advanced GC patients^24^. The sensitivity and specificity of the peritoneal washings samples using OSNA were 83.3% and 87.8%, respectively. These results indicated that intraoperative OSNA assay may serve as a valuable alternative to conventional cytology. Due to its objectiveness and reproducibility, OSNA seems to be a reliable quantitative method of FCCs assessment in the peritoneal fluid^24^.

This study aimed to evaluate the prognostic value of intraoperative peritoneal lavage OSNA assay in advanced GC patients.

## Results

Among the 82 patients included in the study, 44 (53.7%) were male, and 38 (46.3%) were female with a median age of 65 years (range 40–84). According to Lauren classification, there were 35 (42.7%) intestinal, 16 (19.5%) mixed, 22 (26.8%) diffuse type tumours, whereas 9 (11.0%) cases were not classified. Most of the patients included in the study (62.2%) had pT3/4 tumours. Additionally, 45 (54.9%) patients had lymph node metastases (pN+) and 11 (13.4%) patients had limited peritoneal metastases (pM1). The majority (75.6%) of the patients received neoadjuvant CTH. Proximal, distal, and total gastrectomy with a corresponding extent of D1 + /2 lymphadenectomy has been performed in 17.1%, 43.9%, and 39.0%, respectively. Twenty-five (30.5%) patients obtained positive results by OSNA assay, whereas 6 (7.3%) patients were positive by conventional cytology. There were two cases in which the cytology was positive, and OSNA assay was negative. One patient died as a result of postoperative complications, and the other was alive at the time of the last follow up. For OSNA positive patients, the values range from 24.6 to 71,000 cCP/µl. The characteristics of patients included in the study are shown in Table 1. Comparison of OSNA assessment results depending on demographic and clinical variables distribution was presented in Supplementary Table 1. In patients with lower (y)pT stages significantly more often OSNA negative results were observed ((y)pT0; (y)pT1; (y)pT2: 100%; 75%; 72.7%, respectively), while more frequent positive results were noted in patients with the (y)pT4b stage (71.4%) (p = 0.0292). In (y)pM1 patients compared to those classified as (y)pM0, positive OSNA assessment results were observed significantly more often (72.7% vs 23.9%; p = 0.0035). In patients with lower (y)pTNM stages significantly more often OSNA negative results were observed (0; IA; IB: 100%; 75%; 66.7%, respectively), while more frequent positive results were noted in patients in the IV stage (72.7%) (p = 0.0186).

**Table 1:** Patients characteristics.

Variable	Study group (n = 82)	Cytology + (n = 6)	OSNA + (n = 25)	Cytology + /OSNA + (n = 4)
**Sex**				
Men	44 (53.7%)	3 (50%)	15 (60%)	2 (50%)
Women	38 (46.3%)	3 (50%)	10 (40%)	2 (50%)
**Age**				
Median (range)	65 (40–84)			
< 65 years	41 (50%)	3 (50%)	13 (52%)	2 (50%)
≥ 65 years	41 (50%)	3 (50%)	12 (48%)	2 (50%)
**Lauren’s type**				
Intestinal	35 (42.7%)	1 (16.7%)	9 (36%)	1 25%)
Mixed	16 (19.5%)	2 (33.3%)	6 (24%)	1 (25%)
Diffuse	22 (26.8%)	2 (33.3%)	8 (32%)	1 (25%)
Unknown	9 (11.0%)	1 (16.7%)	2 (8%)	1 (25%)
**(y)pT**				
0	8 (9.8%)	–	–	–
1	12 (14.6%)	–	3 (12%)	–
2	11 (13.4%)	–	3 (12%)	–
3	29 (35.4%)	1 (16.7%)	12 (484%)	1 (25%)
4a	15 (18.3%)	3 (50%)	2 (8%)	–
4b	7 (8.5%)	2 (33.3%)	5 (20%)	3 (75%)
**(y)pN**				
0	37 (45.1%)	–	10 (40%)	–
1	13 (15.9%)	–	2 (8%)	–
2	12 (14.6%)	1 (16.7%)	3 (12%)	1 (25%)
3a	15 (18.3%)	3 (50%)	7 (28%)	2 (50%)
3b	5 (6.1%)	2 (33.3%)	3 (12%)	1 (25%)
**(y)pM**				
0	71 (86.6%)	–	17 (68%)	–
1	11 (13.4%)	6 (100%)	8 (32%)	4 (100%)
**(y)pTNM stage**				
0	8 (9.8%)	–	–	–
IA	12 (14.6%)	–	3 (12%)	–
IB	6 (7.3%)	–	2 (8%)	–
IIA	10 (12.2%)	–	5 (20%)	–
IIB	12 (14.6%)	–	1 (4%)	–
IIIA	6 (7.3%)	–	2 (8%)	–
IIIB	11 (13.4%)	–	2 (8%)	–
IIIC	6 (7.3%)	–	1 (4%)	–
IV	11 (13.4%)	6 (100%)	9 (36%)	4 (100%)
**Neoadjuvant chemotherapy**				
Yes	62 (75.6%)	3 (50%)	20 (80%)	3 (75%)
No	20 (24.4%)	3 (50%)	5 (20%)	1 (25%)
**No. of neoadjuvant chemotherapy cycles**				
Median (range)	4 (1–8)	N/a	N/a	N/a
**Tumour regression grade**				
1	6 (9.8%)	–	–	–
2	15 (24.6%)	–	6 (%)	–
3	25 (41%)	2 (66.7%)	7 (28%)	2 (%)
4	15 (24.6%)	1 (33.3%)	6 (40%)	1 (%)
**Type of gastrectomy**				
Proximal	14 (17.1%)	1 (7.1%)	3 (31.6%)	1 (25%)
Distal	36 (43.9%)	–	10 (36.8%)	–
Total	32 (39%)	5 (15.6%)	12 (31.6%)	3 (75%)
**Cytology**				
Positive	6 (7.3%)	N/a	4 (16%)	N/a
Negative	76 (92.7%)		21 (84%)	
**OSNA assay**				
Positive	25 (30.5%)	4 (66.7%)	N/a	N/a
Negative	57 (69.5%)	2 (33.3%)		

*N/a* not applicable, *(y)pT* (post neoadjuvant) pathological primary tumour stage, *(y)pN* (post neoadjuvant) pathological nodal stage, *(y)pM* (post neoadjuvant) pathological distant metastasis stage, *(y)pTNM* (post neoadjuvant) pathological tumour, *node*, metastasis, *OSNA* One-Step Nucleic Acid Amplification Assay.

### Survival analysis

**Figure 1:**
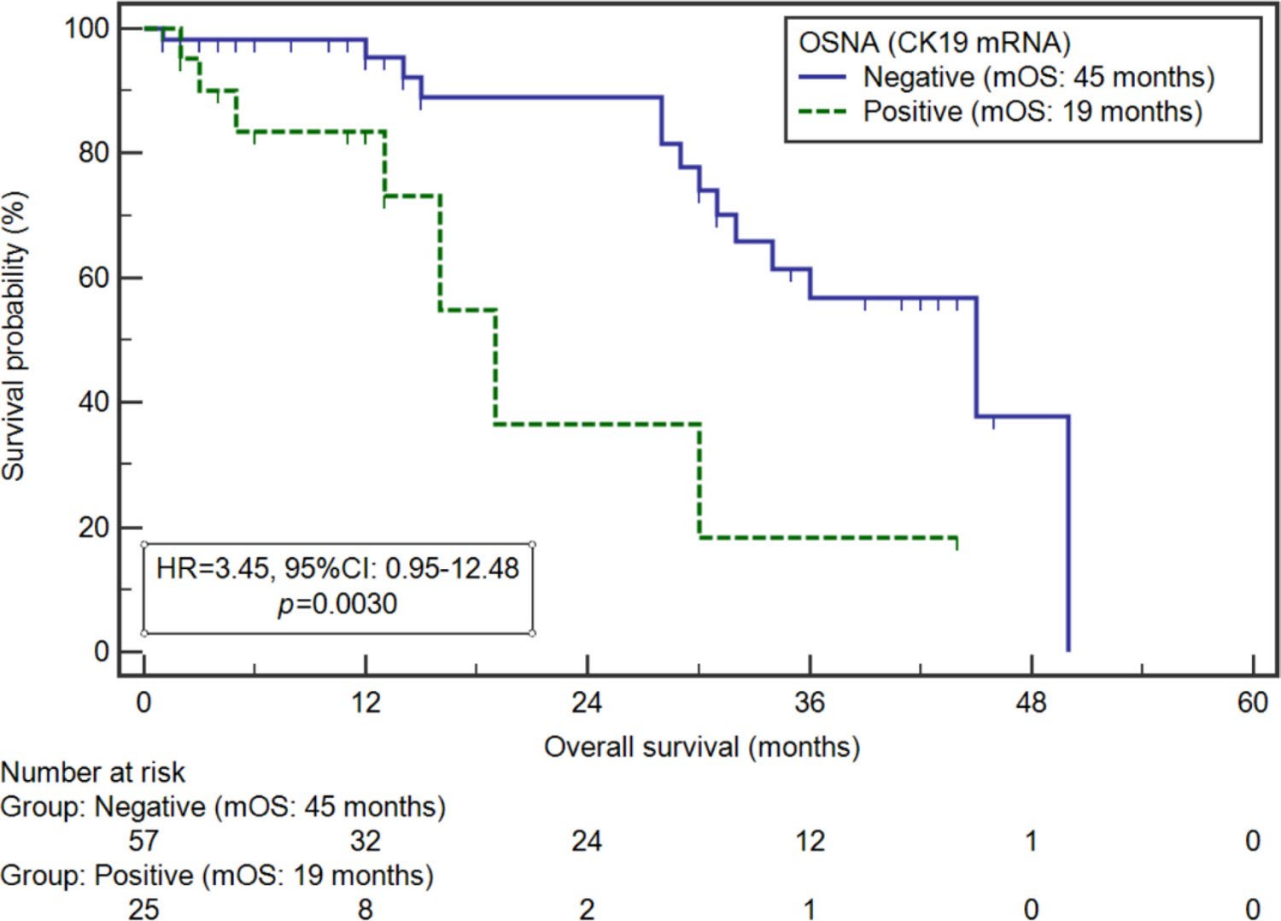
Kaplan–Meier curves representing survival probability depending on OSNA assay status in patients with advanced GC.

Median OS (mOS) for OSNA positive and negative patients were 19 and 45 months, respectively (HR 3.45, 95% CI 0.95–12.45, p = 0.0030) (Fig. 1). Whereas mOS of CY1 and CY0 patients were 50 and 36 months, respectively (HR 0.84, 95% CI 0.22–3.29, p = 0.7595).

**Figure 2:**
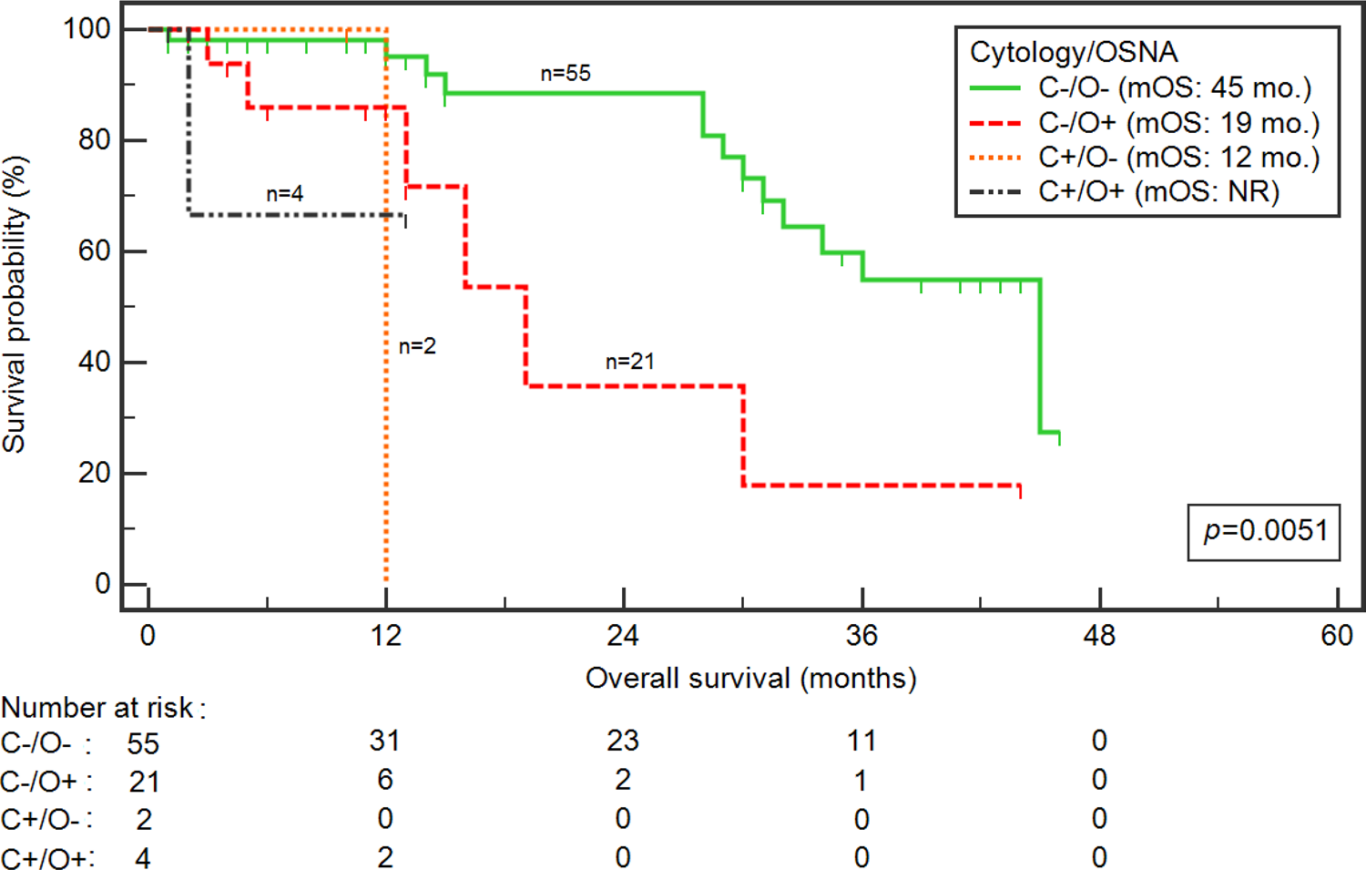
Kaplan–Meier curves representing survival probability depending on combination of cytology and OSNA assay status in patients with advanced GC. *C* cytology, *mo.* months, *NR* not reach, *O* OSNA.

We found statistically significant survival differences after combined cytology assessment and OSNA assay (p = 0.0051) (Fig. 2). Interestingly, a significantly higher risk of death was noted in patients with negative cytology and positive OSNA assay than double-negative patients (19 vs 45 months; HR 3.36, 95% CI 0.84–13.35; *p* = 0.0074).

### Univariable analysis

The univariable analysis of the examined variables showed, that significantly higher risk of death was noted in the case of the non-intestinal GC type (median: 31 vs. NR months; HR 2.99, 95% CI 1.25–7.12; p = 0.0303), higher (y)pT stage ((y)pT3-4; median: 30 vs NR months; HR 7.00, 95% CI 2.94–16.63; p = 0.0017), lymph node involvement ((y)pN -; median: 30 vs 45 months; HR 5.25, 95% CI 2.33–13.09; p = 0.0002), higher (y)pTNM stage (IIIB-IV; median: 29 vs 45 months; HR 3.29, 95% CI 1.33–8.18; p = 0.0027) and lack of tumour regression (TRG3 and TRG4; median: 30 vs NR months; HR 4.22, 95% CI 1.59–11.14; p = 0.0327), and positive result of OSNA assay (19 vs.45 months; HR 3.45, 95% CI 0.95–12.48; p = 0.0030).

### Multivariable analysis

Multivariable analysis confirmed an independent, unfavourable prognostic value of higher (y)pT stage (ypT3-4; HR 7.47, 95% CI 1.72–32.48; p = 0.0076), lymph node involvement (HR 6.18, 95% CI 1.97–19.44; p = 0.0019), higher (y)pTNM stage (IIIB-IV; HR 3.06, 95% CI 1.21–7.76; p = 0.0191) and positive result of OSNA assay (HR 3.10, 95% CI 1.22–8.54; p = 0.0298). Detailed data on the survival analysis in the study group was presented in Table 2.

**Table 2:** Univariable and multivariable analysis of overall survival.

Variable	mOS (months)	Univariable		Multivariable	
		HR [95% CI]	*p*	HR [95% CI]	*p*
**Sex**					
Women	34	1.01 [0.43–2.40]	0.9792	1.18 [0.46–3.01]	0.7287
Men	45				
**Age**					
< 65 years	36	0.92 [0.39–2.17]	0.8365	1.01 [0.40–2.50]	0.9899
≥ 65 years	NR				
**Lauren histological type**					
Intestinal vs	NR	2.99 [1.25–7.12]	0.0303*	2.41 [0.76–7.60]	0.1350
Diffuse/Mixed	31				
**(y)pT**					
0–2	NR	7.00 [2.94–16.63]	0.0017*	7.47 [1.72–32.48]	0.0076*
3–4	30				
**(y)pN**					
N−	45	5.25 [2.33–13.09]	0.0002*	6.18 [1.97–19.44]	0.0019*
N+	30				
**(y)pM**					
0	45	1.85 [0.55–6.24]	0.1371	1.35 [0.40–4.51]	0.6264
1	30				
**(y)pTNM stage**					
0-IIIA	45	3.29 [1.33–8.18]	0.0027*	3.06 [1.21–7.76]	0.0191*
IIIB-IV	29				
**Histopatological grading**					
G2	34	0.37 [0.12–1.16]	0.1688	0.43 [0.09–1.96]	0.2763
G3	NR				
**Neoadjuvant chemotherapy**					
Yes	50	2.13 [0.81–5.64]	0.1763	3.35 [0.71–15.84]	0.1293
No	34				
**Tumour Regression Grade**					
1, 2	NR	4.22 [1.59–11.14]	0.0327*	2.90 [0.62–13.66]	0.1801
3, 4	30				
**Type of gastrectomy**					
Proximal, Distal vs	NR	2.00 [0.84–4.69]	0.1022	1.69 [063–4.51]	0.2994
Total	32				
**Cytology**					
Negative	36	0.84 [0.22–3.29]	0.7595	0.27 [0.03–2.40]	0.2426
Positive	50				
**OSNA assay**					
Negative	45	3.45 [0.95–12.48]	0.0030*	3.10 [1.22–8.54]	0.0298*
Positive	19				

Reference (control) variables were underlined.

*Statistically significant results. *mOS* median overall survival, *HR* hazard ratio, *CI* confidence interval, *(y)pT* (post neoadjuvant) pathological primary tumour stage, *(y)pN* (post neoadjuvant) pathological nodal stage, *(y)pM* (post neoadjuvant) pathological distant metastasis stage, *TNM* tumour, node, metastasis, *G* grade, *OSNA* One-Step Nucleic Acid Amplification Assay, *NR* not reached.

## Discussion

Our study focused on the prognostic value of OSNA assay for detecting single tumour marker (CK19) mRNA in intraoperative peritoneal lavage in advanced GC patients. Multiple studies on genetics biomarkers in peritoneal washings have demonstrated that PCR positive patients have worse OS and earlier peritoneal recurrence than PCR negative patients^25–28^. In the present study, the mOS in OSNA positive patients was significantly lower than in OSNA negative patients. Additionally, OSNA assay on peritoneal washings showed an additional prognostic value to conventional cytology, as reflected in both, univariate and multivariate analysis. Moreover, mixed type GC and nodal involvement were associated with a significant increase in the risk of death. It is known that mixed type carcinomas have more aggressive clinical behaviour correlated with greater tumour size, submucosal and lymphovascular invasion^29^. According to the Japanese classification, mixed histological types exhibit higher lymph node metastasis rates than pure differentiated or undifferentiated types^30^. However, for higher stage cancers, diffuse type may have worse prognosis^31^.

Although the first reports on FCC detection by cytology in GC patients were published nearly 70 years ago^32^, there is no consensus on the management of patients with positive peritoneal cytology. The treatment recommendations range from palliative CTH to neoadjuvant CTH followed by conversion surgery if regression is achieved^6,33^. In the REGATTA trial, gastrectomy followed by CTH did not demonstrate any OS benefit compared with CTH alone in advanced GC patients with a single non-curative factor^5^. However, in patients with GC and CY1 or low-volume peritoneal dissemination, perioperative S-1 based CTH^34^ or hyperthermic intraperitoneal chemotherapy (HIPEC)^35^ or intraperitoneal and intravenous paclitaxel plus S-1^36^ may be considered as an addition to gastrectomy with curative intent.

Patients with negative cytology findings may experience unexpected peritoneal recurrence after surgery^12^. These results indicate the lack of sensitivity on conventional cytology for detecting FCC and predicting peritoneal recurrence. Our findings indicate the additional value of the OSNA assay in detecting FCC in peritoneal washings when conventional cytology is negative. Studies showed that molecular biology techniques increase the sensitivity of FCC detection^37,38^. A positive result of CEA mRNA's cytology and transcription-reverse transcription concentrated reaction (TRC) was observed in 20% and 54% of patients, respectively^38^. Similarly, our study showed that OSNA assay increases the detection of FCC. We found 7.3% patients positive by cytology and 30.5% positive by OSNA examination. The positive result of RT-PCR assay has correlated with poor prognosis and short survival^39^. A recent meta-analysis on the prognostic significance of molecular analysis of peritoneal fluid in GC patients showed that even in CY0 patients, positive molecular status increases the risk of a poor prognosis by over twofold^23^. In our study, twenty-one patients were OSNA positive out of seventy-six CY0 patients. Therefore, nearly one-third of the study group gained additional prognostic information with the OSNA assay for intraperitoneal lavage assessment. Applying SL with intraoperative lavage as part of the diagnostic process in patients with GC may help identify microscopic peritoneal dissemination and choose an appropriate treatment modality^39^. A meta-analysis showed that CY0 before treatment is associated with more prolonged survival than CY1^40^.

Furthermore, it is postulated that cytology should be considered a modifiable factor as a conversion from CY1 into CY0 after systemic CTH is associated with OS improvement^40^. Although CY1 is considered stage IV disease, the prognosis and OS of patients with positive cytology or macroscopic peritoneal metastases are not equivalent^39^. The OS was significantly improved in the solitary CY1 group^40^. Neoadjuvant CTH resulted in the downstaging of the disease in 61% of CY1 patients^39^. On the other hand, nearly 25% of CY0 patients converted into CY1 after receiving neoadjuvant CTH^41^. Prediction of advanced GC patients response to neoadjuvant CTH remains a challenge.

In contrast to conventional cytology, which is based on the assessment of cell morphology, quantitative molecular methods have greater reproducibility and objectivity, favouring their application in laboratory practice^23^. However, several factors can impair the standardisation of a molecular method for routine diagnostic workup. The sensitivity of molecular methods is mostly determined by the marker gene expression level and the relevant background level in peritoneal lavage. According to the human protein atlas^42^, amplified CK-19 mRNA may originate from various cells. Expression at the background level demands a cut-off approach, especially for samples such as intraperitoneal lavage, in debris and various other cells^43^. Additionally, the expression of markers in FCC might differ from the tissue-resident cells or marker concentration may vary within the tissue due to intratumoural heterogeneity^44^. We could not sufficiently establish the specificity of the assay yet. CK-19 mRNA might be produced by other cells in the abdomen and therefore further studies and respective validation are needed. It is postulated that using a panel of various tumour markers may further increase the sensitivity and specificity of molecular detection of FCC^15^. Future studies to determine whether analysis of multiple tumour markers rather than a single gene may improve the diagnostic utility of OSNA examination.

Limitations of our study include the small sample size and relatively short follow-up time. Extended follow-up with larger sample size is essential to assess if OSNA examination of peritoneal washings can be utilised instead of conventional cytology. Another limitation of our study is the heterogeneity of the patient population, which might cause unintended bias. Nevertheless, our findings reveal that positive intraperitoneal OSNA assay is a promising prognosticator of poor survival in GC patients, which may help select patients requiring more aggressive intraperitoneal treatment such as HIPEC or prolonged/modified neoadjuvant systemic therapy.

## Conclusions

Positive OSNA assay in intraperitoneal lavage is a valuable indicator of poor survival in GC patients after multimodal treatment. After further confirmation on a larger sample size, OSNA assay of peritoneal washings could be considered as an adjunct tool to conventional cytology that is the current gold standard to provide precise intraoperative staging and additional prognostic information.

## Methods

This observational study was conducted after obtaining institutional review board approval (Bioethical Committee of Medical University of Lublin, Ethic Code: KE—0254/297/2018). Written informed consent was obtained from the patients in line with the principles outlined in the Declaration of Helsinki. We collected data from database of patients operated on GC between July 2017 and Jun 2021 in the Department of Surgical Oncology, Medical University of Lublin, Poland. Inclusion criteria were: histologically confirmed gastric adenocarcinoma scheduled for gastrectomy either following neoadjuvant CTH or upfront surgery with adjuvant radiochemotherapy. Type of gastrectomy and extent of lymphadenectomy (D1+, D2) was performed at the surgeon’s discretion. The exclusion criteria were no resection: explorative laparotomy (laparoscopy) or any palliative non-resection procedure (bypass, jejunostomy). The study included 82 consecutive patients in whom intraperitoneal lavage was performed after surgical exploration. Patients were staged following the 8th AJCC/TNM classification. Neoadjuvant treatment was received by 75% of patients, therefore the abbreviation (y)pTNM was introduced to emphasize that preoperative therapy did not apply to the entire study group. Modified Becker’s system was used for the pathological evaluation of tumour regression grade (TRG)^45^.

### Intraoperative lavage examination

After laparotomy and thorough exploration of the abdominal cavity, intraoperative lavage with 100 ml of saline solution was performed in all included patients. The washings obtained from the tumour area (50 ml) were divided into two equal parts (25 ml). One was intended for cytological examination, and the other one was centrifuged for 10 min at 1500×*g* in order to obtain cellular sediment. The cell pellet was subjected to OSNA examination.

### OSNA examination

Peritoneal lavage samples for OSNA assay were centrifuged for 10 min at 1500×*g* to obtain a cell pellet. The cellular sediment was stored at − 80 °C until OSNA examination. As we previously described, peritoneal lavage was assessed according to the protocol for OSNA performance^24^. The first step of sample preparation was homogenisation of the cell sediment using LYNORHAG homogenising buffer, pH 3.5 (Sysmex, Kobe, Japan). The process was carried out with a RP-10 homogenizer (Sysmex, Kobe, Japan). One ml of LYNORHAG homogenising buffer, pH 3.5 (Sysmex, Kobe, Japan) was added to cellular pellet. The cells with homogenising buffer were transferred to LYNOPREP tube (Sysmex, Kobe, Japan), and homogenized for 90 s. at 10,000 rpm. In this process, CK-19 mRNA was released from the tumour cells. Then 1 ml of homogenate was centrifuged for 1 min. at 12.200 rpm. After that 20 µl of centrifuged OSNA lysate was transferred to a RD Sample Vial (Sysmex, Kobe, Japan) with pre-set 180 µl of LYNORHAG. A ready-to-use LYNOAMP gene amplification reagent kit (Sysmex, Kobe, Japan) was used to perform the RT-LAMP reaction. A volume of 2 µl of previously prepared sample, 20 µl of CK19 primer solution, and 3 µl of enzyme solution were pipetted by the pipetting unit of RD-210 gene amplification detector (Sysmex, Kobe, Japan) into the respective detection cell in the reaction block. The reaction solution was mixed and the reaction block subsequently heated up to reaction temperature of 64 °C. A reverse transcription reaction was followed by the targeted gene amplification reaction. Amplification time took 11 min per sample. The RT-LAMP technique amplifies a targeted mRNA with high specificity, efficiency, and rapidity under isothermal conditions^46–48^. Reaction mixture contained tree pairs of primers: 5′-GGAGTTCTCAATGGTGGCACCAACTACTACACGACCATCCA-3′ (CK19 forward inner primer), 5′-GTCCTGCAGATCGACAACGCCTCCGTCTCAAACTTGGTTCG-3′ (CK19 reverse inner primer), 5′-TGGTACCAGAAGCAGGGG-3′ (CK19 forward outer primer), 5′-GTTGATGTCGGCCTCCACG-3′ (CK19 reverse outer primer), 5′-AGAATCTTGTCCCGCAGG-3′ (CK19 forward loop primer), and 5′-CGTCTGGCTGCAGATGA-3′ (CK19 reverse loop primer)^49^. The RT-LAMP method measured the time required to exceed a defined threshold of turbidity caused by magnesium pyrophosphate, a by-product of the reaction. The change in turbidity correlates with the amount of CK19 mRNA calculated from the standard curve value. All results below 250 cCP/µL (a cut-off set in RD-210 analyzer to distinguish positive and negative lymph node) were calculated based on the current standard curve. As we published before, the cut-off value for distinguishing positive and negative cases for peritoneal washings samples was set at 24.6 cCP/µL^25^. At this cut-off value for peritoneal washings samples, the sensitivity and specificity were 83.3% and 87.8%, respectively^24^. The RD-210 gene amplification detector allows for faster examination of a larger number of samples. Converted values from the new system are expressed with new unit, cCP/µL^50^. The procedure of OSNA assessment can be completed within 30 min, so that it meets intraoperative requirements.

### Conventional cytology

Cytological examination after hematoxylin and eosin (H&E) and mucicarmine staining were performed by an experienced cytopathologist from our Hospital Pathology Department.

### Statistical analysis

The MedCalc v.15.8 software (MedCalc Software, Belgium) was used to perform the statistical analysis. Due to the lack of studies evaluating the prognostic value of OSNA assessment performed in peritoneal washings in patients with GC, we retrospectively calculated sample size in the acquired data set. The post-hoc calculation was based on comparing percentages of patients with 3-year survival (median OS in the study group was equal to 36 months) and primary endpoint—OSNA assessment result (positive or negative). Most medical studies consider a p-value below 0.05 to reject the null hypothesis, thus type I error (alpha) of 0.05 value was used. In the case of type II error, we set a cut-off of beta on 0.2 to achieve 80% of statistical power. Considering the percentage of patients with 3-year survival in OSNA positive (12.5%) and negative cases (54.2%), as well as the ratio of sample sizes in compared groups (5:1), the minimal study group was estimated as 75 patients. Moreover, we have additionally considered approximately 10% of missing data. Thus, we estimated that the minimal number of 82 patients should be included. Due to the lack of a normal data distribution (assessed by the D'Agostino-Pearson test), the median and the range were used to present the concentration and dispersion of the data. Categorized or dichotomized variables were expressed as numbers and percentages. Overall survival (OS) was defined as the time from the date of surgery to the date of the patients death or the date of the last follow-up. None of the patients was lost from follow-up (median was 12 months). Survival data were updated in May 2021. In univariable OS analysis, we used the log-rank test to calculate the proportional hazard ratio (survival curves were generated with the use of Kaplan–Meier estimation method). In multivariable analysis, Cox logistic regression models were used. All statistically significant results of the univariable analysis were considered potentially valuable for multivariable analysis. However, since not all patients underwent neoadjuvant chemotherapy, this variable was not included in the multivariable model. Moreover, the backward elimination method revealed that only (y)pTNM and OSNA assay were significantly related to survival and thus, only those variables were finally used for adjustment in multivariable analysis. The only exceptions were models for ypT and ypN in which results were adjusted by (y)pN and (y)pT, respectively (instead of (y)pTNM) as they are elements of this composite measure. In all analyses, we used two-tailed tests. All results with a p-value below 0.05 were considered to be statistically significant.

## Supplementary Information

Supplementary Table 1.

## Data Availability

All data generated or analysed during this study are included in this published article and its supplementary information files.
