# Author Correction: Prognostic value of molecular cytology by one‑step nucleic acid amplifcation (OSNA) assay of peritoneal washings in advanced gastric cancer patients

**DOI:** 10.1038/s41598-023-28808-5

**Published:** 2023-01-27

**Authors:** Katarzyna Gęca, Magdalena Skórzewska, Karol Rawicz‑Pruszyński, Radosław Mlak, Katarzyna Sędłak, Zuzanna Pelc, Teresa Małecka‑Massalska, Wojciech P. Polkowski

**Affiliations:** 1 Department of Surgical Oncology, Medical University of Lublin, Radziwiłłowska 13 St., 20‑080 Lublin, Poland; 2 Department of Human Physiology, Medical University of Lublin, Radziwiłłowska 11 St., 20‑080 Lublin, Poland

Correction to: *Scientific Reports* 10.1038/s41598-022-16761-8, published online 21 July 2022

In the original version of this article Katarzyna Gęca and Magdalena Skórzewska were omitted as equally contributing authors.

The original Article has been corrected.

