# Supplementary material for: Prognostic value of molecular cytology by one-step nucleic acid amplification (OSNA) assay of peritoneal washings in advanced gastric cancer patients
Source: Sci Rep. 2022 Jul 21;12:12477. doi: 10.1038/s41598-022-16761-8 (PMC9304381; doi:10.1038/s41598-022-16761-8)
Supplement: Supplementary file 1 — Supplementary Table 1. [file 41598_2022_16761_MOESM1_ESM.pdf]

Supplementary table 1. Comparison of OSNA assessment results depending on distribution of demographic and clinical variables.

**Variable**	**OSNA**<br>negative<br>**(n=57)**	**OSNA**<br>positive<br>**(n=25)**	***p***
**Sex**<br>Men<br>Women	29 (65.9%)<br>28 (73.7%)	15 (34.1%)<br>10 (26.3%)	0,6016
**Age**<br><65 years<br>≥65 years	 <br>28 (68.3%)<br>29 (70.7%)	 <br>13 (31.7%)<br>12 (29.3%)	1.0000
**Lauren`s type**<br>Intestinal<br>Mixed<br>Diffuse<br>Unknown	26 (74.3%)<br>10 (62.5%)<br>14 (63.6%)<br>9 (%)	9 (25.7%)<br>6 (37.5%)<br>8 (36.4%)<br>-	0.1671
**(y)pT**<br>0<br>1<br>2<br>3<br>4a<br>4b	7 (100%)<br>9 (75%)<br>8 (72.7%)<br>17 (58.6%)<br>13 (86.7%)<br>2 (28.6%)	-<br>3 (25%)<br>3 (27.3%)<br>12 (41.4%)<br>2 (13.3%)<br>5 (71.4%)	0.0292*
**(y)pN**<br>0<br>1<br>2<br>3a<br>3b	27 (73%)<br>11 (84.6%)<br>9 (75%)<br>8 (53.3%)<br>2 (40%)	10 (27%)<br>2 (15.4%)<br>3 (25%)<br>7 (46.7%)<br>3 (60%)	0.2238
**(y)pM**<br>0<br>1	54 (76.1%)<br>3 (27.3%)	17 (23.9%)<br>8 (72.7%)	0.0035*
**(y)pTNM stage**<br>0<br>IA<br>IB<br>IIA<br>IIB<br>IIIA<br>IIIB<br>IIIC<br>IV	8 (100%)<br>9 (75%)<br>4 (66.7%)<br>5 (50%)<br>11 (91.7%)<br>4 (66.7%)<br>9 (81.8%)<br>4 (66.7%)<br>3 (27.3%)	-<br>3 (25%)<br>2 (33.3%)<br>5 (50%)<br>1 (8.3%)<br>2 (33.3%)<br>2 (18.2%)<br>2 (33.3%)<br>8 (72.7%)	0.0186*
**Neoadjuvant chemotherapy**<br>Yes<br>No	 42 (67.7%)<br>15 (75%)	 20 (32.3%)<br>5 (25%)	0.7385
**Tumour Regression Grade**<br>1<br>2<br>3<br>4	6 (100%)<br>9 (60%)<br>18 (72%)<br>9 (60%)	-<br>6 (40%)<br>7 (28%)<br>6 (40%)	0.2614

**Type of gastrectomy**<br>Proximal<br>Distal<br>Total	11 (78.6%)<br>26 (72.2%)<br>20 (62.5%)	3 (21.4%)<br>10 (27.8%)<br>12 (37.5%)	0.4943
**Cytology**<br>positive<br>negative	2 (33.3%)<br>55 (72.4%)	4 (66.7%)<br>21 (27.6%)	0.1238

Abbreviations: (y)pT – (postneoadjuvant) pathological primary tumour stage, (y)pN - (postneoadjuvant) pathological nodal stage, (y)pM - (postneoadjuvant) pathological distant metastasis stage, (y)pTNM - (postneoadjuvant) pathological tumour, node, metastasis, OSNA - One-Step Nucleic Acid Amplification Assay; * - statistically significant results.
